# Whole-Genome Sequencing of a Human Clinical Isolate of *emm28 Streptococcus pyogenes* Causing Necrotizing Fasciitis Acquired Contemporaneously with Hurricane Harvey

**DOI:** 10.1128/genomeA.01269-17

**Published:** 2017-11-09

**Authors:** S. Wesley Long, Priyanka Kachroo, James M. Musser, Randall J. Olsen

**Affiliations:** a Center for Molecular and Translational Human Infectious Diseases Research, Department of Pathology and Genomic Medicine, Houston Methodist Research Institute and Houston Methodist Hospital, Houston, Texas, USA; b Department of Pathology and Laboratory Medicine, Weill Cornell Medical College, New York, New York, USA

## Abstract

We discovered an *emm28 Streptococcus pyogenes* isolate causing necrotizing fasciitis in a patient exposed to the floodwaters of Hurricane Harvey in the Houston, TX, metropolitan area in August 2017. The Oxford Nanopore MinION instrument provided sufficient genome sequence data within 1 h of beginning sequencing to close the genome.

## GENOME ANNOUNCEMENT

*Streptococcus pyogenes* (group A streptococcus [GAS]) causes infections ranging in severity from innocuous pharyngitis (“strep throat”) to life-threatening necrotizing fasciitis (flesh-eating disease) (1).

We sequenced the genome of strain HarveyGAS to closure using the Ligation sequencing kit 1D, R9.4 flow cell, and Oxford Nanopore Technologies MinION Mk-Ib sequencer. Short-read sequence data were also generated using the Illumina Nextera XT kit and a MiSeq instrument. The Unicycler hybrid assembler version 0.4.1 achieved complete closure of the genome using a combination of the Illumina and Oxford Nanopore data (2). The HarveyGAS chromosome is 1,852,980 bp. No extrachromosomal plasmids were detected.

To taxonomically classify the HarveyGAS strain, we first used the Oxford Nanopore antimicrobial resistance workflow, which includes the 1D What’s In My Pot (WIMP) workflow for identification. After only 1 h of analysis, sufficient reads had been collected to clearly identify strain HarveyGAS as GAS, most likely type *emm28*. We confirmed the *emm28* prediction using the Illumina reads, SRST2, and a custom *emm*-type database (3). No acquired antimicrobial resistance genes were identified by the Oxford Nanopore antimicrobial resistance workflow or SRST2, consistent with the antimicrobial resistance phenotype determined by automated methods in our clinical microbiology laboratory. Mapping sequencing reads against the original *emm28* reference genome (strain MGAS6180) showed that HarveyGAS differs by 140 single-nucleotide polymorphisms (SNPs) from this reference genome. Of note, the strain lacks the 6180.2 prophage, a 42.3-kb segment of DNA that encodes SpeK and SlaA (4). The remaining prophage and prophage remnants (6180.1, 6180.3, and 6180.4) found in MGAS6180 are present in the HarveyGAS genome.

The occurrence of this *emm28* GAS strain in a wound in a patient exposed to the floodwaters of Hurricane Harvey helps illustrate that even during periods of significant natural disasters, common pathogens present in common ways. Despite a clinical suspicion of *Vibrio* species or other aquatic-associated pathogens, GAS was the agent in this case of a severe necrotizing infection. *emm28* strains are among the more common GAS serotypes to cause invasive infections in the United States (5). The ability of the Oxford Nanopore MinION system to rapidly sequence the genome of this isolate and provide information in real time allowed for sufficient reads to be recovered and analyzed and allowed closure of the genome while the sequencer was still running. In this context, this technology is a substantial advance over conventional next-generation sequencing methods that use fixed run times and lengthy postrun bioinformatics to obtain FASTQ files prior to downstream analysis (6).

### Accession number(s).

The accession number for the HarveyGAS closed genome is CP023769. The accession number for the MGAS6180 reference genome is CP000056.
